# Influence of Environmental Factors on the Germination of *Urena lobata* L. and Its Response to Herbicides

**DOI:** 10.1371/journal.pone.0090305

**Published:** 2014-03-21

**Authors:** Tahir Hussain Awan, Bhagirath Singh Chauhan, Pompe C. Sta. Cruz

**Affiliations:** 1 Weed Science, Crop and Environmental Sciences Division, International Rice Research Institute (IRRI), Los Baños, Laguna, Philippines; 2 Crop Science Cluster, College of Agriculture, University of Philippines Los Baños, Laguna, Philippines; University of Vigo, Spain

## Abstract

*Urena lobata* is becoming a noxious and invasive weed in rangelands, pastures, and undisturbed areas in the Philippines. This study determined the effects of seed scarification, light, salt and water stress, amount of rice residue, and seed burial depth on seed germination and emergence of *U. lobata*; and evaluated the weed's response to post-emergence herbicides. Germination was stimulated by both mechanical and chemical seed scarifications. The combination of the two scarification methods provided maximum (99%) seed germination. Germination was slightly stimulated when seeds were placed in light (65%) compared with when seeds were kept in the dark (46%). Sodium chloride concentrations ranging from 0 to 200 mM and osmotic potential ranging from 0 to −1.6 MPa affected the germination of *U. lobata* seeds significantly. The osmotic potential required for 50% inhibition of the maximum germination was −0.1 MPa; however, some seeds germinated at −0.8 MPa, but none germinated at −1.6 MPa. Seedling emergence and biomass increased with increase in rice residue amount up to 4 t ha^−1^, but declined beyond this amount. Soil surface placement of weed seeds resulted in the highest seedling emergence (84%), which declined with increase in burial depth. The burial depth required for 50% inhibition of maximum emergence was 2 cm; emergence was greatly reduced (93%) at burial depth of 4 cm or more. Weed seedling biomass also decreased with increase in burial depth. Bispyribac-sodium, a commonly used herbicide in rice, sprayed at the 4-leaf stage of the weed, provided 100% control, which did not differ much with 2,4-D (98%), glyphosate (97%), and thiobencarb + 2,4-D (98%). These herbicides reduced shoot and root biomass by 99–100%.

## Introduction

*Urena lobata* L., an annual weed, belongs to the family Malvaceae. This weed is known by many local names in different countries, for example, Afulut Gad (Philippines), China pink flowered Chinese Burr or Xiao fan tian hua (China), Africain Jute (Africa), Kongojute (Germen), Bachita (India), Aguaxima (Brazil), Aramina or Cadillo (Spanish), Ke hoa dao (Vietnam), and *Urena lopastnaia* (Russia) [1]. *U. lobata* originated in Asia and has invaded most of the tropical and subtropical area of the world [2]. According to the United States Department of Agriculture (USDA) plant profile database, *U. lobata* has infested areas in Puerto Rico, Florida, Louisiana, the U.S. Virgin Islands, and New South Wales [2]. At present, *U lobata* is a common intruder in natural open areas, pastures, and gardens. It can grow over a wide altitude range; from near sea-level to about 1000-m above sea level. This weed usually grows in rainforests along roads, monsoon forests, and in disturbed areas. From these observations, the Exotic Pest Plant Council of Florida promptly placed *U. lobata* on the list of nuisance plants under Category II in 1999, which implies that the plant is increasing in number but has not yet been established as ecologically harmful [3].

During the 1970s and 1980s, the new *U. lobata* cultivar Ex-Mokwa was introduced and began to be grown as a fiber crop in Sierra Leone [4] and experimentally grown for many years [5]. This species is also used for its medicinal properties in Malaysia and Java. The leaves and barks are used as a contraceptive, and leaf and root extracts are used to prepare herbal medicines [6]. In some parts of Africa, its leaves and flowers are used as food during famine. *U. lobata* had limited success as a crop, but its cultivation resulted in its rigorous infestation as a weed in many areas. Rangelands, poorly managed disturbed areas, pastures, road sides, and eroded areas are easily infested by *U. lobata*. It can thrive on a wide range of soil types and can grow up to a height of 3 m with a basal diameter of 7 cm [7]. Throughout the year, *U. lobata* produces flowers and fruits with up to 600 seeds per plant [8]. *U. lobata* fruits are produced in about 1 cm-diameter capsules covered by straight trichomes that end in about 4–6 small hooks. These hooks cause the fruits to stick on horses' manes and tails, sheep's hair, clothes, and other similar objects that come into contact with plants.

Under field conditions, *U. lobata* seeds revealed dormancy due to an impermeable seed coat (testa) [9]. This is the main cause of poor germination under field conditions [5]. Low or irregular germination, owing to seed dormancy, has been reported by Kirkby [10]. To improve germination, mechanical scarification through removal of carpels and by rubbing the seeds in sand, have been suggested [11]. Sulfuric acid scarification has also been found successful in overcoming seed dormancy [9]. The focus of most of the research work done on *U. lobata* in the past was to improve plant characteristics including growth, seed yield, and fiber yield [5], [8], [12]. From research conducted on seed germination, it has been speculated that scarification of seeds is necessary to break seed dormancy [5]. The cause of poor germination is the high levels of dormancy due to an impermeable seed coat. However, the role of light, water stress, salt stress, and soil burial depth on seed germination is not well understood for the species. *U. lobata* is becoming a noteworthy weed in many parts of the world, especially in rice fields of Bangladesh, India, Indonesia, Philippines, Thailand, Vietnam [13] and Florida pastures [14]. Research is needed to better understand the seed biology of *U. lobata* and its control with herbicides. A better understanding of the environmental factors that affect the germination of *U. lobata* seeds may help in explaining its fast proliferation in various ecosystems.

Seed germination is usually influenced, directly or indirectly, by many environmental factors such as temperature, moisture, and light [15]. For example, temperature can affect germination by regulating the enzymes activities involved in germination and by promoting or inhibiting the synthesis of hormones that affect seed dormancy [15]. Germination response of seed to fluctuating temperatures is a mechanism by which seeds detect gaps in vegetation canopies and depth of burial in soil [16]. Requirement of light for germination of a weed species means that the species will germinate only when its seeds are on or near the soil surface. Similarly, the ability of a species to germinate under moisture- and salt-stressed conditions will allow that species to take advantage of conditions that suppress the germination of other species. Information on the effect of seed burial depth on seedling emergence may indicate the effectiveness of tillage operations in weed control. Crop residue on the soil surface may be helpful in weed suppression [17] and can be incorporated as a tool in integrated weed management plans. Seedling emergence may be inhibited or stimulated by crop residue, and will depend on the quantity and quality of crop residue and the biology of the weed species involved [18].

The herbicide 2,4-D is used for broadleaf weed control in agricultural and nonagricultural situations. Glyphosate is usually used as a non-selective herbicide to control all weeds before crop-sowing. However, glyphosate is marginally effective on some weeds, such as the *Ipomoea* species [19]. Little is known about the effect of herbicides on *U. lobata*, and, therefore, there is a need to evaluate the performance of available herbicides against it.

The objectives of this study were to determine the optimum time for soaking seeds in sulfuric acid to break seed dormancy; assess the effect of light, water stress, salt stress, soil burial depth, and quantity of rice straw on *U. lobata* seed germination and seedling emergence; and evaluate the performance of commonly available post-emergence herbicides on *U. lobata*.

## Materials and Methods

All experiments in the laboratory and screenhouse were laid out in a randomized complete block design with four replications. Each experiment was conducted twice.

### Seed collection and germination test

Seeds of *U. lobata* were collected in March 2011 at the International Rice Research Institute (IRRI) farm in Los Baños, Laguna, Philippines. The first two authors work at this institute, and thus no permission was needed to collect the weed seeds. They also confirmed that the study did not involve endangered or protected species.

Seeds collected from at least 150 plants were bulked and cleaned. Experiments were conducted at laboratory and screenhouse facilities of IRRI. Seed germination of *U. lobata* was determined by placing 25 seeds evenly spaced in a 9 cm-diameter Petri dish containing two layers of Whatman No. 1 filter paper (Whatman International Ltd., Maidstone, U.K.) moistened with 5 ml of distilled water or a treatment solution. Parafilm was used to wrap the Petri dishes to reduce water loss. Seeds were scarified with concentrated sulfuric acid (98% H_2_SO_4_) for 30 minutes (min). Seeds were washed with running tap water for 5 min after treating with sulfuric acid and before being subjected to various treatments. Seeds were placed in an incubator at fluctuating day/night temperatures of 30/20°C in a light/dark regime, unless otherwise specified. This temperature regime was selected because it has been found appropriate for several weed species in the Philippines. The photoperiod was set at 12 h to coincide with the high-temperature photoperiod. Fluorescent lamps were used to produce a photosynthetic photon flux density (PPFD) of 88 micro mol m^−2^ s^−1^.

Seed germination was counted daily up to 15 d after placement in the incubator except for the continuous dark treatment, in which germination was counted once at 15 DAS. Germinated seeds (with the radicle visible) were removed from the Petri dishes at each counting. Water was added to the filter paper as needed. Non-germinated seeds were subjected to a simple pressure test to evaluate viability. White firm embryos were considered viable while brown soft embryos were considered nonviable [15].

### Effect of seed scarification on germination

To establish whether germination in *U. lobata* is inhibited by an impermeable seed coat, seeds were placed in concentrated sulfuric acid (98%) for 5, 10, and 30 min. In another treatment, seeds were mechanically scarified first by using two rubber stoppers, and then soaking the seeds in sulfuric acid for 30 min. Non-treated seeds were used as a control treatment. Seeds were incubated as described above, and germination was determined.

### Effect of light on germination

The effect of light on germination was determined by incubating scarified seeds of *U. lobata* at 30/20°C temperatures (alternating day/night) in both light/dark (12 h/12 h) and continuous (24 h) dark regimes. To maintain the conditions of complete darkness, Petri dishes were wrapped with a double layer of aluminum foil. Other experimental conditions were constant, as previously stated in the germination protocol. As weed seeds do not undergo continuous light and constant temperature conditions under normal conditions in nature [18], these conditions were not included in the experimental variables.

### Effect of salt stress on germination

Seed germination as affected by salt stress was investigated by placing scarified seeds of *U. lobata* in dishes each containing a 5 ml of solution of 0, 25, 50, 100, and 200 mM sodium chloride (NaCl) (Mallinckrodt Baker Inc., Phillipsburg, NJ). These ranges of NaCl reflect salinity levels occurring in the tropics [20].

### Effect of osmotic stress on germination

The effect of osmotic stress on seed germination was determined by incubating seeds in solutions with osmotic potentials of 0, −0.1, −0.2, −0.4, −0.8, and −1.6 MPa, which were prepared by dissolving 0, 28.9, 40.9, 57.8, 81.8, and 115.6 g of polyethylene glycol 8000 (Sigma-Aldrich Co., St. Louis, MO) in 300 ml of distilled water [21].

### Effect of amount of rice residue on emergence and biomass of seedlings

Fifty scarified seeds of *U. lobata* were sown on the soil surface in plastic pots. Rice residue (leaves and stems of the rice variety NSICRc222) was spread on the soil surface at rates equivalent to 0, 1, 2, 4, and 6 t ha^−1^. The amounts of rice straw used in this study reflect the amount of straw produced in low-yield rainfed environments and high-yield irrigated environments. The condition of the soil and the pots, emergence counting, and harvesting in this experiment were done as described above for the seed burial experiment.

### Effect of seed burial depth on emergence and biomass of seedlings

The effect of seed burial depth on seedling emergence was investigated in a screenhouse. Fifty scarified seeds of *U. lobata* were covered with soil to depths of 2, 4, 6, and 8 cm or placed on the soil surface in plastic pots (15 cm in diameter). The soil had 22% sand, 38% silt, 40% clay, 35% organic carbon, 0.107% N, 0.121% Kjeldahl N, 43 mg kg^−1^ soil available P, 1.26 meq 100^−1 ^g soil available K, and a pH of 6.0. Soil used for this experiment was collected from rice fields, autoclaved, and passed through a 3-mm sieve. Pots were watered initially with an overhead mist sprinkler and later sub-irrigated. Plants were watered throughout the study as needed to maintain the optimal moisture level for seed germination. Seedlings were considered emerged when a cotyledon was visible on the soil surface. Emergence was counted at 3-day intervals up to 30 days after sowing (DAS). Emerged seedlings were counted and harvested at 30 DAS. After harvesting, sample plants were oven-dried at 70°C for 72 h to obtain dry biomass. After oven-drying, the biomass of plants was measured.

### Efficacy of herbicides

To evaluate the effect of recommended rates of herbicides on survival, shoot biomass, root biomass, and control of *U. lobata*, 20 scarified seeds were planted on the soil surface in plastic pots and covered with a thin layer of soil. The condition of the soil and pots used in this experiment were the same as that described for the seed burial experiment. Seedlings were thinned to 8 plants per pot at 5 DAS and later maintained at this density. Post-emergence herbicides 2,4-D ester at 0.5 kg ai ha^−1^; glyphosate at 1 kg ai ha^−1^; bispyribac-sodium at 0.03 kg ai ha^−1^; thiobencarb + 2,4-D at 0.8 kg ai ha^−1^; and fenoxaprop-p-ethyl + ethoxysulfuron at 0.045 kg ai ha^−1^ were applied using a Research Track Sprayer (DeVries Manufacturing, Hollandale, MN) that delivered 210 L ha^−1^ spray solution at a spray pressure of 140 kPa. Flat-fan nozzles (Teejet 80015) were used in the sprayer. Herbicides were sprayed on the seedlings of *U. lobata* at the 4- and 6-leaf stages. The difference between the two leaf stages was 9 days. Herbicides were not sprayed in the control treatments for each leaf stage. Seedling survival was determined at 25 days after herbicide application, with the criterion being the appearance of a new sprouting leaf. After counting the surviving plants, these were removed from the pots. Soil was washed off through a steel strainer. Plants were separated into shoots and roots and oven-dried at 70°C for 72 h to obtain dry biomass weight.

### Statistical analyses

In the laboratory and screenhouse, each replication was arranged on different shelves in the incubator or on different benches in the screenhouse. Different shelves in the incubator or different benches in the screenhouse may experience yet varying conditions, and therefore each replication was considered as a block. Data were subjected to ANOVA and means were separated using least significant difference (LSD) at 5% level of significance. No interaction was detected between the treatments and experimental runs; therefore, data were pooled over the runs for analysis (GenStat 8.0). Before statistical analysis, homogeneity of variance was visually confirmed by inspecting the residuals.

Germination, emergence, and biomass data were analyzed using regression analysis. Germination resulting from scarification and salt- and water-stress treatments, and emergence resulting from different burial depths and residue amounts were modeled using a functional three-parameter sigmoid function. The fitted model was:
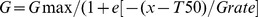
where *G* is the cumulative percentage germination at time *x, G_max_* is the maximum germination (%), *T_5_*_0_ is the time (d) required for 50% of maximum germination, and *G_rate_* indicates the slope. An exponential decay curve in the form of
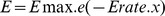
was fitted to seedling emergence (%) or seedling biomass (g) obtained at different depths, where *E* represents cumulative emergence (%) and biomass (g) at depth *x, E_max_* is the maximum emergence or biomass, and *E_rate_* indicates the slope. Parameter estimates for each model were compared using their standard errors [22]–[24]. Survival and weed control data were transformed before statistical analysis using arcsine transformation. The transformation did not improve the results; therefore, the nontransformed values were used for analysis.

## Results and Discussion

### Effect of seed scarification on germination

**Figure 1:**
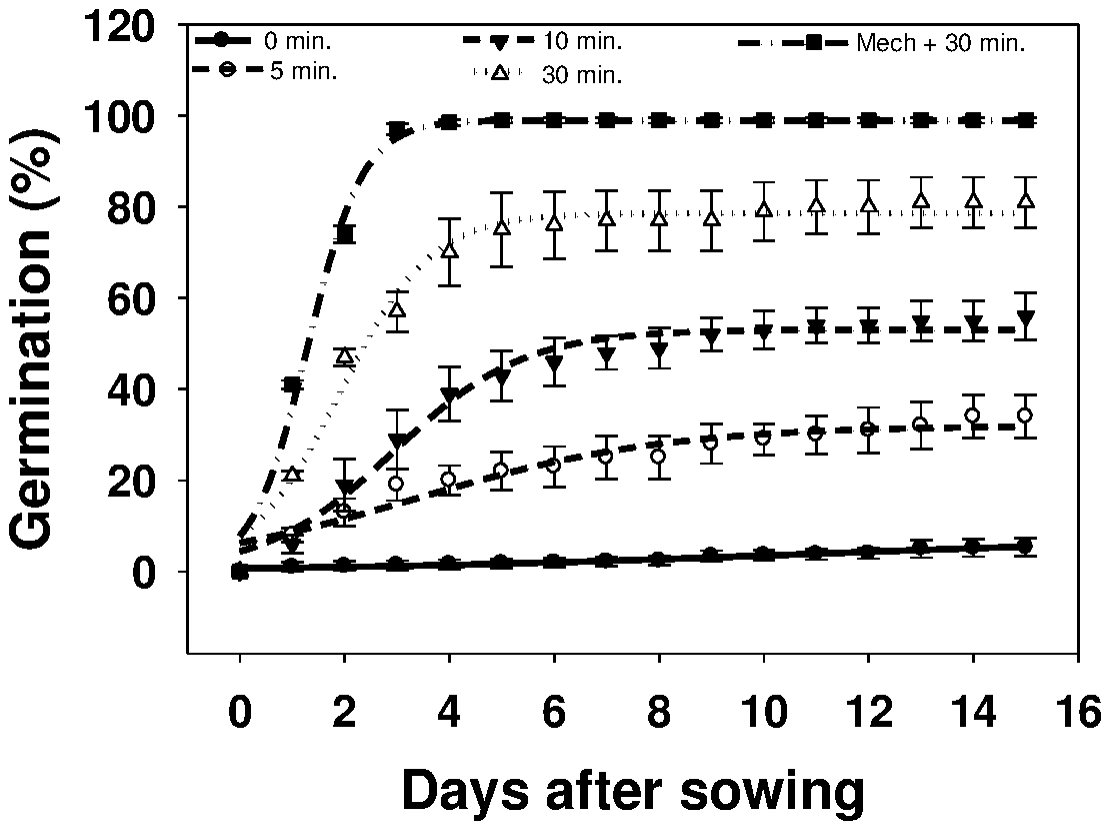
Effect of seed-soaking time in concentrated sulfuric acid (0, 5, 10, and 30 min) and mechanical scarification + soaking time (Mech + 30 min) on germination of *Urena lobata* seed, modeled with the use of equation *G* = *G_max_*/(1+e [−(*x*−*T_50_*)/*G_rate_*]. Estimated parameters are given in Table 1. Vertical bars represent standard error of the mean.

This study included the effect of mechanical and chemical scarifications on the germination of *U. lobata*. Increased soaking time in concentrated sulfuric acid was found to improve the germination percentage (Figure 1). Maximum germination (80%) was achieved at 30 min of soaking in sulfuric acid compared to the control (3%). Mechanical scarification plus 30 min of soaking in sulfuric acid resulted in the highest germination rate (99%) (Table 1). Similar observations were reported in an earlier study [14], in which mechanical and chemical scarification enhanced germination rate of *U. lobata* by 42 and 78%, respectively.

**Table 1:** Effect of seed-soaking time in concentrated sulfuric acid (0, 5, 10, and 30 min) and mechanical scarification + soaking time in sulfuric acid (Mech + 30 min) on germination of *Urena lobata* seed.

Soaking time in sulfuric acid (min)	*G_max_*	*G_rate_*	*T_50_*	*R^2^*
0	3.0 (1.5)	4.67 (0.73)	10.90 (1.96)	0.98
5	32.0 (1.4)	2.39 (0.44)	3.36 (0.43)	0.94
10	53.1 (0.9)	1.23 (0.14)	2.95 (0.15)	0.98
30	78.6 (1.1)	0.87 (0.10)	1.92 (0.11)	0.98
Mechanical + 30	99.9 (0.8)	0.53 (0.05)	1.30 (0.05)	0.99

Parameter estimates [*G_max_*, maximum germination (%); *T_50_*, time to reach 50% of maximum germination (days); and *G_rate_*, slope] of a three-parameter sigmoid model, 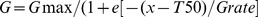, fitted to seed germination data in Figure 1. Values in parentheses represent standard error of the mean.

Mechanical scarification was laborious and time consuming, and may not be done uniformly on a large quantity of seeds. Thus, *U. lobata* seeds were chemically scarified (30 min of soaking) for all the subsequent experiments. Non-germinated seeds in all treatments were tested through the pressing method as described earlier [15] and were found to be viable. These results are consistent with earlier findings that sulfuric acid scarification was effective in breaking seed dormancy in *U. lobata* [17] and *Ipomoea triloba* L. [25].

Seed dormancy of *U. lobata* was mainly caused by its hard seed coat. Physical dormancy is widespread among Malvaceae species [5] and is the most ubiquitous form of dormancy among plant species [27]. Mechanical or chemical scarification can break, scratch, or soften seed coats, which effectively release dormancy in *U. lobata* seeds. Based on previous research on *U. lobata* [14], it was pragmatic that the germination percentage of intact seeds (without removing husk) was less than 2%. The low percentage of germination was attributed to the enclosure of the seed inside a bur, which resulted in low germination rates [28]. Removal of the husk increased germination slightly (10%), while water leaching inside resulted in a nearly 20% germination.

Research suggests that seed coat-imposed dormancy may be a significant cause for low germination. The hard seed coat helps *U. lobata* persist for a long period of time in the soil. When dormancy is released, seeds start to germinate. The results of the present study suggest that *U. lobata* seeds are less likely to germinate in the field unless scarified. It may thus persist in the soil for a long period of time, causing a setback in future crops, if scarification does not take place. Under natural environmental conditions, scarification usually occurs due to soil burial of seed [28], [29], microbial attack [29], acute changes in temperature which cause contraction and expansion of the seed coat) [30], [31], humid atmospheric conditions [32], [33], passage through the digestive system of animals [34], [35], flora and fauna actions, burning of vegetation and fire release the dormancy of hard seed especially if fires coincide with wet and hot conditions [36], [37], [38], and scratching by soil particles probably during tillage operations [28]. However, the effects of these factors are not yet known in the case of *U. lobata*.

### Effect of light on germination

The average germination percentage of *U. lobata* seed was 65% (±2.5%) in light/dark and 46% (±6.1%) in continuous dark conditions (data not shown). The results suggest that light is not a prerequisite for the germination of *U. lobata* and that its seeds can germinate without light. This may explain why *U. lobata* is often found under tree canopies within pastures, rangelands, and natural areas. Other plant species of the Malvaceae family, such as *Sida spinosa* L., do not require light for germination [26].

### Effect of salt stress on germination

**Figure 2:**
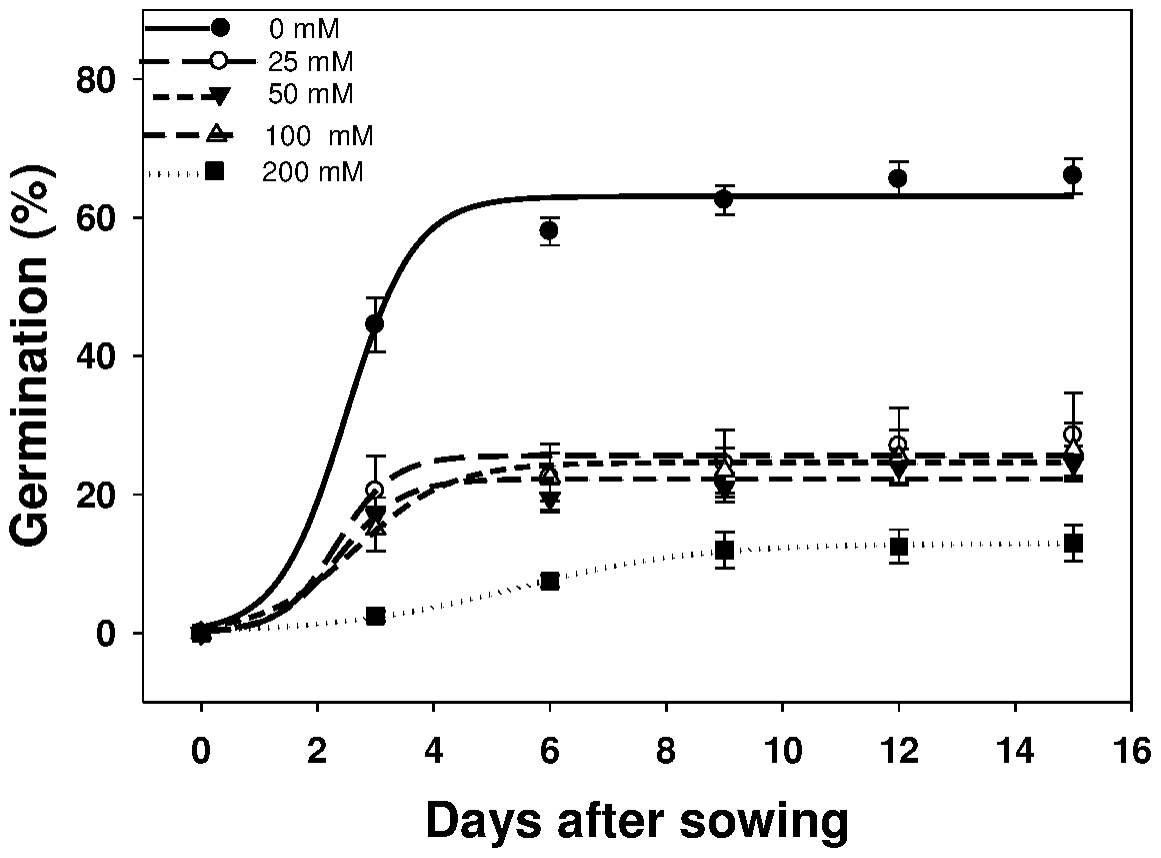
Effect of sodium chloride concentrations (mM) on the germination of *Urena lobata* seeds, incubated at 30/20°C in light/dark, modeled with the use of equation *G* = *G_max_*/(1+e [−(*x*−*T_50_*)/*G_rate_*]. Estimated parameters are given in Table 2. Vertical bars represent standard error of the mean.

Salt concentration affected the germination of *U. lobata* greatly (Figure 2). Seeds had the highest cumulative germination (63%) in the nonstressed treatment (0 mM NaCl); cumulative germination declined with increasing salt concentration. Germination of *U. lobata* was at 22% at NaCl concentration of 100 mM, and decreased drastically to 13% at 200 mM concentration. It is evident from the data that salt stresses significantly delayed the start of germination and reduce germination percentage. The time to inhibit 50% of the maximum germination (*T_50_*) increased with increase in the level of salt stress (Table 2).

**Table 2:** Effect of sodium chloride concentrations (mM) on the germination of *Urena lobata* seeds, incubated at 30/20°C in light/dark.

NaCl (mM)	*G_max_*	*G_rate_*	*T_50_*	*R^2^*
0	63.1 (1.8)	0.59 (0.47)	2.49 (0.43)	0.99
25	25.6 (1.3)	0.48 (1.03)	2.34 (1.45)	0.98
50	24.6 (0.9)	0.80 (0.44)	2.68 (0.28)	0.98
100	22.3 (1.2)	0.54 (0.86)	2.37 (1.03)	0.96
200	12.9 (0.3)	1.55 (0.15)	5.42 (0.17)	0.998

Parameter estimates [*G_max_*, maximum germination (%); *T_50_*, time to reach 50% of maximum germination (days); and *G_rate_*, slope] of a three-parameter sigmoid model, 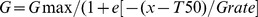, fitted to seed germination data in Figure 2. Values in parentheses represent standard error of the mean.

Similar results on the effect of varying NaCl concentrations were reported for *Amaranthus spinosus* L. and *A. viridis* L. [24], *I. triloba* [25], *Chromolaena odorata* (L.) King and H.E. Robins and *Tridax procumbens* L. [22], and *Digitaria ciliaris* (Retz.) Koel. and *D. longiflora* (Retz.) Pers. [23]. In Asia, a significant area (21.5 M ha) has been documented to have salt-affected soil [39]. Soil with more than 100 mM concentration of NaCl is considered as having a high level of salinity. Rice, a staple food of people in the tropics, is considered a salt-sensitive crop [40]. Salinity stress, even at 40 mM NaCl, can affect flooded rice [41]. Some species in the Asteraceae, Fabaceae, and Poaceae families were reported as able to adapt to saline conditions [20], [22], [23]. In this study, *U. lobata* germinated even at 200 mM NaCl. This characteristic may allow *U. lobata* to have a distinct advantage over other crops, such as rice, in these saline environments.

### Effect of osmotic stress on germination

**Figure 3:**
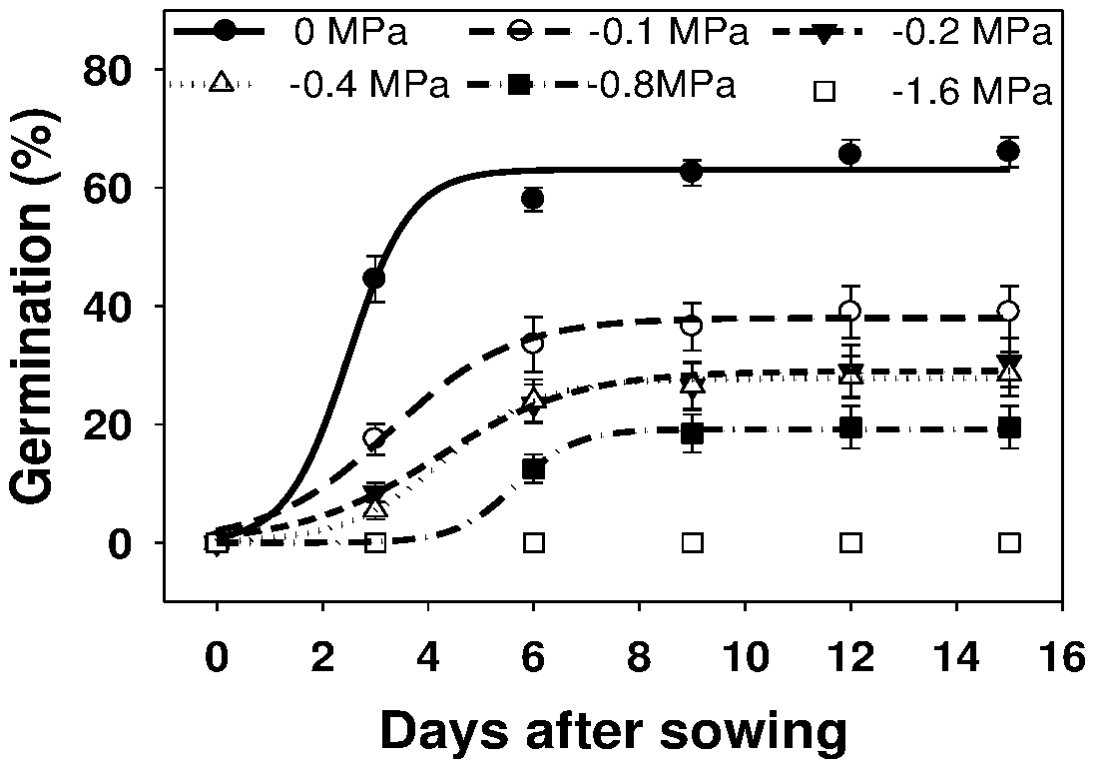
Effect of different osmotic potential (MPa) on germination of *U. lobata* seed, in 3-day intervals, incubated at 30/20°C in light/dark, modeled with the use of equation *G* = *G_max_*/(1+e [−(*x*−*T_50_*)/*G_rate_*]. Estimated parameters are given in Table 3. Vertical bars represent standard error of the mean.

Germination of *U. lobata* was affected greatly by increasing water stress (Figure 3). The maximum germination of *U. lobata* at 0 MPa was 63%. An osmotic potential of −0.1, −0.2, −0.4, −0.8, and −1.6 MPa reduced germination of *U. lobata* to 38, 29, 28, 19, and 0%, respectively (Figure 3; Table 3). Time to 50% germination (*T_50_*) increased with increase in degree of osmotic stress.

**Table 3:** Effect of different osmotic potential (MPa) on germination of *U. lobata* seed, incubated at 30/20°C in light/dark conditions.

Osmotic Potential (MPa)	*G_max_*	*G_rate_*	*T_50_*	*R^2^*
0	63.1 (1.8)	0.59 (0.47)	2.49 (0.43)	0.988
−0.1	38.0 (1.1)	1.16 (0.25)	3.29 (0.24)	0.987
−0.2	29.0 (1.0)	1.30 (0.23)	4.19 (0.29)	0.990
−0.4	27.8 (0.5)	0.93 (0.09)	4.30 (0.15)	0.998
−0.8	19.2 (0.3)	0.57 (0.27)	5.65 (0.17)	0.998
−1.6	0	0	0	0

Parameter estimates [*G_max_*, maximum germination (%); *T_50_*, time to reach 50% of maximum germination (days); and *G_rate_*, slope] of a three-parameter sigmoid model, 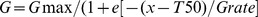, fitted to seed germination data in Figure 3. Values in parentheses represent standard error of the mean. No germination occurred at −1.6 MPa.

The results of our study suggest that germination of *U. lobata* is favored by a moist environment and that seeds can remain ungerminated in drought conditions. This could be a valuable tactic of *U. lobata*, allowing for an increased ability to delay germination until favorable conditions occur, which thus prolongs the existence of these seeds in the seed bank. Similar results were reported by Wang and colleagues, wherein *U. lobata* seed germination was significantly affected by increasing water stress. Germination percentage was at 86% in deionized water and, as water stress is increased, germination declined [14].

Seed germination is a process of growth of a previously dormant or inactive seed starting with the imbibitions of water [42]. Seed imbibition rate and germination normally decrease as the water potential of the surrounding decreases, and the critical hydration level for seed germination is species-specific [43].

Based on the present study, germination of *U. lobata* cannot occur when water potential is equal or lower than −1.6 MPa, which is the case for soils that are dry or have high salt levels. The tolerance level of a weed species to water stress is related to its ecological habitat. Species, such as *Eupatorium compositifolium* Walt., when in dry or well-drained terrestrial habitats, can usually germinate at low moisture or water potential [44]. Conversely, species that require moist soils for germination, such as *A. spinosus* and *A. viridis*, can germinate only when water potential is at −0.6 MPa or higher [24], [45]. Our data support the findings reported for *I. triloba* [25], *A. spinosus* and *A. viridis* [24], [45], in which germination decreased with decrease in water potential. At 30/20°C alternating temperatures, no seeds germinated at water potential of −0.6 MPa and below. Germination of *A. retroflexus* L. was also sensitive to water availability, with final germination being reduced by 50% at −0.4 MPa compared to 0 MPa [46]. *U. lobata* seeds are sensitive to water stress and may not germinate during the dry season in well-drained soils. Rainfall is typically low during April and May in the Philippines, which may delay the germination of *U. lobata* until the rainy season starts in June.

### Effect of amount of rice residue on emergence and biomass of seedlings

**Figure 4:**
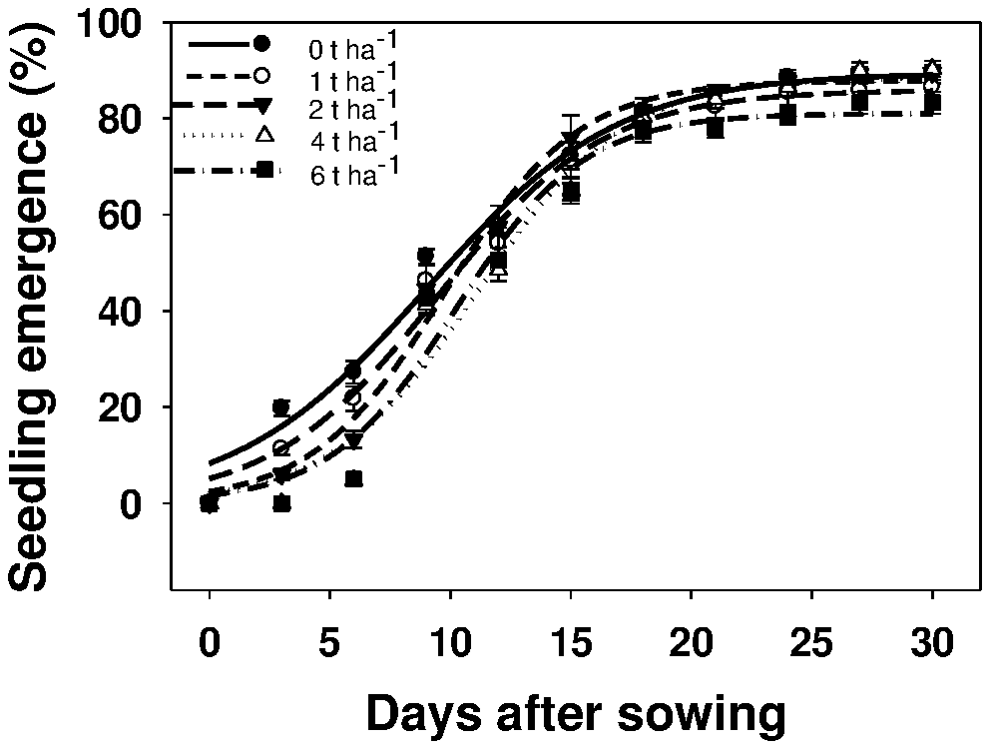
Effect of rice residue amount (t ha^−1^) on emergence of *Urena lobata* at various days after sowing, modeled with the use of equation *G* = *G_max_*/(1+e [−(*x*−*T_50_*)/*G_rate_*]. Parameter estimates are given in Table 4. Vertical bars represent standard error of the mean.

Seedling emergence continued until 27 DAS and subsequently became constant (Figure 4). The emergence of *U. lobata* was not influenced by the amount of residue tested, and it varied from 81 to 90% (Figure 4; Table 4). Seedling biomass was not influenced by the amount of rice residue, and was in the range of 5.4–6.2 g pot^−1^ (data not shown). Similar results were reported for *A. viridis* [24]. Contrary to our findings on *U. Lobata*, however, the emergence of several weed species (e.g., *C. odorata*, *Tridax procumbens*, *A. spinosus*, and *A. viridis*) were reduced by the addition of crop residue [22], [24].

**Table 4:** Effect of rice residue amount (t ha^−1^) on seedling emergence of *Urena lobata*.

Residue quantity (t ha^−1^)	*G_max_*	*G_rate_*	*T_50_*	*R^2^*
0	89.5 (2.7)	3.97 (0.49)	9.04 (0.55)	0.98
1	85.9 (1.8)	3.44 (0.32)	9.47 (0.37)	0.99
2	87.9 (1.7)	2.76 (0.28)	9.79 (0.32)	0.99
4	88.5 (3.4)	3.03 (0.52)	11.17 (0.61)	0.98
6	81.0 (3.0)	2.65 (0.50)	10.25 (0.58)	0.98

Parameter estimates [*G_max_*, maximum germination (%); *T_50_*, time to reach 50% of maximum germination (days); and *G_rate_*, slope] of a three-parameter sigmoid model, 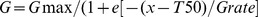, fitted to seedling emergence data in Figure 4. Values in parentheses represent standard error of the mean.

### Effect of seed burial depth on emergence and biomass of seedlings

**Figure 5:**
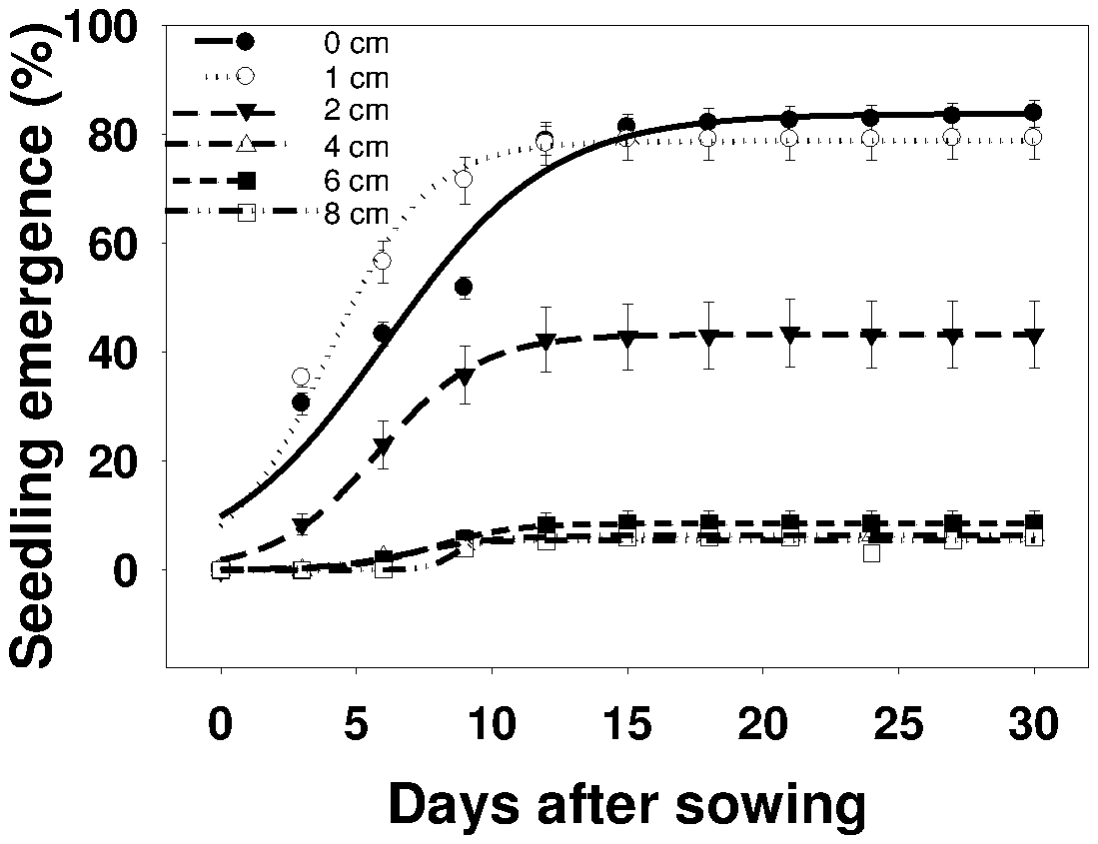
Effect of seed burial depth (cm) on seedling emergence of *Urena lobata* at various days after sowing, modeled with the use of equation *G* = *G_max_*/(1+e [−(*x*−*T_50_*)/*G_rate_*]. Estimated parameters are given in Table 5. Vertical bars represent standard error of the mean.

The emergence of *U. lobata* was markedly affected by seed burial depth (Figure 5). Seeds placed on the soil surface had an emergence percentage of 84% that decreased with increase in burial depth, i.e., 79% at 1 cm and 50% at 2 cm. Increase in burial depth to 6 and 8 cm resulted in 9 and 5% emergence, respectively. With increase in soil burial depth, there was a resulting increase in time (d) to reach 50% of the maximum emergence, except in the treatment where seeds were placed on the soil surface. For example, time to reach 50% emergence was 4, 6, 7, 8, and 9 days at seed burial depths of 1, 2, 4, 6, and 8 cm, respectively (Table 5).

**Table 5:** Effect of seed burial depth (cm) on seedling emergence of *Urena lobata* Parameter estimates [*G_max_*, maximum germination (%); *T_50_*, time to reach 50% of maximum germination (days); and *G_rate_*, slope] of a three-parameter sigmoid model, 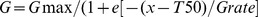, fitted to seedling emergence data in Figure 5.

Soil depth (cm)	*G_max_*	*G_rate_*	*T_50_*	*R^2^*
0	83.8 (2.7)	3.02 (0.65)	6.09 (0.60)	0.97
1	78.7 (1.5)	1.84 (0.27)	3.96 (0.30)	0.98
2	43.2 (0.3)	1.87 (0.10)	5.82 (0.11)	0.99
4	6.3 (0.1)	1.53 (0.17)	7.05 (0.20)	0.99
6	8.5 (0.1)	1.45 (0.08)	7.87 (0.09)	0.99
8	5.3 (0.4)	0.45 (3.38)	8.52 (3.65)	0.89

Values in parentheses represent standard error of the mean.

**Figure 6:**
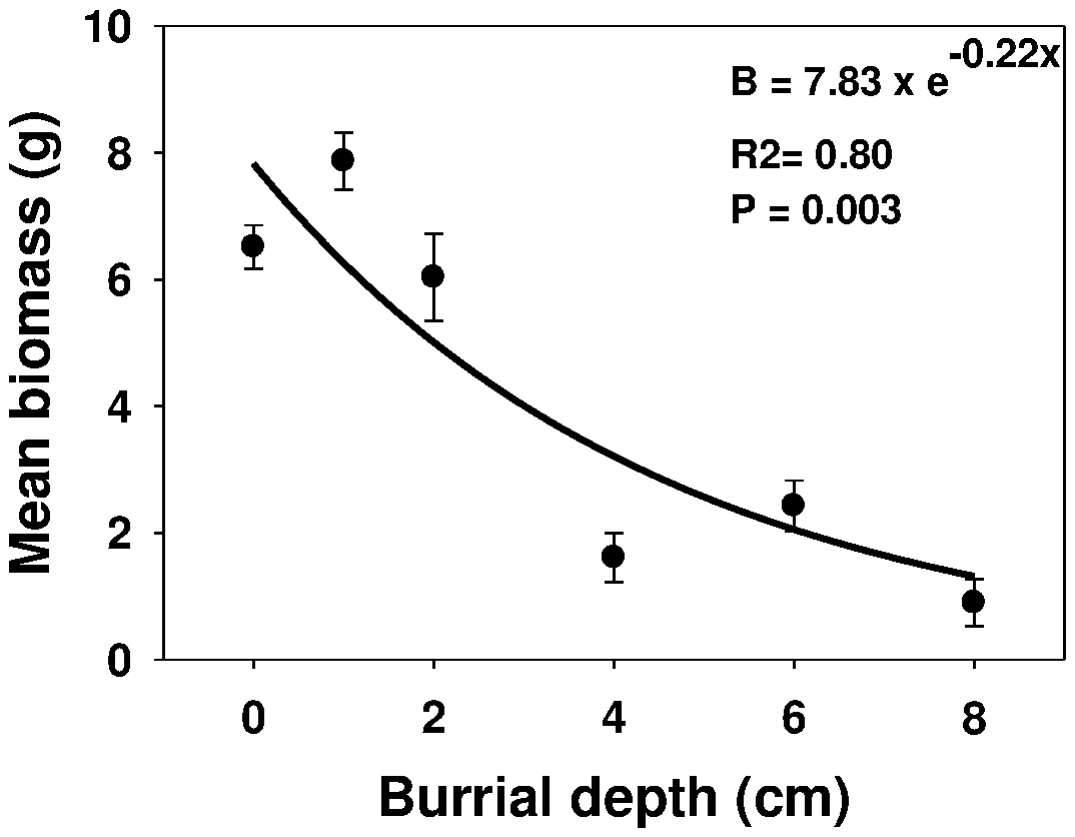
Effect of different seed burial depth (cm) on seedling biomass (g pot^−1^) of *Urena lobata* at 30 days after sowing, modeled with the use of equation *E* = *E_max_ x e* ^(-Erate.x)^. Vertical bars represent standard error of the mean.

Biomass was lower (6.5 g pot^−1^) for seedlings emerging from the soil surface compared with those emerging from 1 cm burial depth (7.9 g pot^−1^) (Figure 6). However, seedling biomass decreased with burial depth beyond 1 cm. Biomass was only 0.9 g pot^−1^ for seedlings emerging from a depth of 8 cm.

These results are in agreement with reported findings on *I. triloba* [25]. *U. lobata* seeds are most likely to germinate within 2 cm of the soil surface, but low germination rate (6%) was observed at burial depth of 8 cm. A similar germination percentage (84%) was reported by Wang and workers when *U. lobata* seeds were placed on the soil surface [14]. Probably at deeper depths, light and seed size are usually the limiting factors for seedling emergence. Light penetration is generally limited to the first few millimeters below the soil surface [47]. However, the present study found that light is not a prerequisite for *U. lobata* seed germination. In previous studies, reduced emergence of several weed species due to increased burial depth were reported [22], [24].

Fluctuating temperatures are more likely to promote germination of small-seeded species than of large-seeded species [16]. Seeds of small weed species are more likely to be buried [48], [49], and their seedlings are less likely to emerge from deeper soil, from where seedlings of large seeds can emerge [50]. Thus, depth sensing is much more imperative for small seeds than for large seeds. Small-seeded species, such as *A. spinosus* and *A. viridis*, may have limited reserves to support germination and seedling emergence, thus limiting the depth from which their seedlings can emerge [24]. Conversely, larger seeds usually have higher reserves of carbohydrate and are capable of emerging from greater burial depths [17], such as in the case of *Sicyos angulatus* L. that could emerge even from a depth of 16 cm [51]. The seed diameter of *U. lobata* is about 2 mm, compared to that of *S. angulatus* that is about 5 mm. *U. lobata* seedlings thus failed to emerge when buried too deep. *U. lobata* seedlings were able to emerge from a depth of 6 cm, suggesting the possibility of an escape mechanism when treated with pre-emergence herbicides. Another possible reason for germination inhibition caused by burial depth may be the secondary dormancy imposed by the interaction between soil gases and seed metabolism [52], [53]. The lack of emergence from deeply buried seeds could be attributed to hypoxia and low rates of gaseous diffusion deep in the soil [47], [54].

The results of the present study suggest that farming practices that enable shallow burial of weed seeds will only suppress the emergence of *U. lobata* seedlings but may not provide complete control of *U. lobata* emergence. On the one hand, deep-tillage operations may be needed to overturn the soil and bury *U. lobata* seeds very deep, from where its seedlings are unable to emerge. On the other hand, succeeding tillage operations may bring viable weed seeds near the soil surface and cause these to germinate and re-infest the succeeding crop. To cope with weed infestation, it is necessary to understand seed viability in the soil seed bank.

### Efficacy of herbicides

*Urena lobata* seedlings did not survive when sprayed with bispyribac-sodium at the 4-leaf stage, resulting in 100% weed control. Similar weed control was achieved with 2,4-D, glyphosate, and thiobencarb + 2,4-D (Table 6). These herbicides reduced the shoot and root biomass by 99–100%. At the 6-leaf stage, the above-mentioned herbicides, except bispyribac-sodium, had similar effects but with little decline in weed control (80%). Bispyribac-sodium at this leaf stage provided poor control (11%). As far as root and shoot biomass is concerned, other herbicides reduced biomass by 97% while bispyribac-sodium reduced biomass by 82%. Similar results from the use of 2,4-D and glyphosate were reported for *I. triloba* [25]. Percentage control and survival data suggest that fenoxaprop-p-ethyl + ethoxysulfuron provided poor control (9 and 0% at 4- and 6-leaf stages, respectively).

**Table 6:** Effect of post-emergence herbicides on survival (%), shoot and root biomass (g pot^−1^), and control (%) of *Urena lobata* when sprayed at 4- and 6-leaf stages of the weed.

	Rate	4-leaf stage				6-leaf stage			
Herbicide		Survival	Shoot biomass	Root biomass	Control	Survival	Shoot biomass	Root biomass	Control
	kg ha^−1^	%	g pot^−1^	g pot^−1^	%	%	g pot^−1^	g pot^−1^	%
Control	-	100	4.342	1.647	0	100	6.971	2.053	0
2,4-D ester	0.50	1.6	0.006	0.001	98.4	17.2	0.229	0.051	82.8
Bispyribac-sodium	0.03	0	0	0	100	89.1	1.336	0.329	10.9
Fenoxaprop+ ethoxysulfuron	0.045	90.6	1.014	0.307	9.4	100	2.447	0.809	0
Glyphosate	1.00	3.1	0.014	0.003	96.9	12.5	0.117	0.021	87.5
Thiobencarb + 2,4-D	0.80	1.6	0.008	0.005	98.4	18.8	0.218	0.072	81.2
LSD_0.05_		7.88	0.1727	0.0748	7.88	10.74	0.3376	0.1309	10.74
*P*		<0.001	<0.001	<0.001	<0.001	<0.001	<0.001	<0.001	<0.001

Manual control of a weed plant that has a fibrous root system, such as *U. lobata*, is very difficult, time consuming, and expensive. Therefore, farmers have to rely on chemicals to control *U. lobata*. Knowledge of herbicides that are effective against *U. lobata* is very important for its control. Based on the results of this study, it can be concluded that if *U. lobata* infests upland rice, farmers can easily control this weed through the application of 2,4-D or thiobencarb + 2,4-D. These herbicides can provide excellent control of *U. lobata* and the control is comparable to glyphosate application. Glyphosate application remains as the ideal control option for *U. lobata* when there is no rice crop in the field.
